# Socio-Psychological and Design Features Related to Transport Choices: A Focus Group Research in the Metropolitan Area of Cagliari (Sardinia, Italy)

**DOI:** 10.3389/fpsyg.2021.808509

**Published:** 2022-01-20

**Authors:** Sara Manca, Francesca Ausilia Tirotto, Nicola Mura, Ferdinando Fornara

**Affiliations:** ^1^Department of Education, Psychology, Philosophy, University of Cagliari, Cagliari, Italy; ^2^School of Psychology, University of Plymouth, Plymouth, United Kingdom

**Keywords:** perceived behavioral control, social norms, safety, environmental stress, public transport, emotions

## Abstract

Due to the environmental and health impact of the private transport sector, social scientists have largely focused on psychosocial and contextual factors associated with people's choice over transport means. This study aims to contribute to this line of research by applying a user-centered approach, with the objective of taking into account the specific environmental and social context of the metropolitan area of Cagliari city (Sardinia, Italy). To accomplish this aim, four groups of people were matched according to their shared starting point: car users vs. public transport users (Study 1), and light-rail users vs. non-light-rail users (Study 2). Groups were interviewed using a focus group method. Participants were invited to discuss their everyday travel experiences and to exchange their ideas on different sustainable (e.g., bicycles and public transport) and less sustainable (i.e., private cars) means of transport. Both consolidated drivers/barriers in the field of environmental psychology (e.g., perceived behavioral control, social norms) and public transportation design features (e.g., lighting) have been investigated. Other than highlighting the importance of socio-psychological factors to promote more sustainable transport choices like in previous studies, the present research offers an insight into how these aspects and factors are shaped and experienced in the narratives of residents.

## Introduction

Limiting the use of private cars in favor of more sustainable transport choices, such as public transport and bicycle use, is associated with a range of positive environmental impacts (e.g., Brand et al., 2021) and health benefits (e.g., Patterson et al., 2020). However, the use of private cars rather than public transport still seems the most preferred means of urban transport in the European Union (Eurostat, 2021a). In fact, data pointed out that in the EU, there is a regional average rate of 0.54 passenger cars per inhabitant (Eurostat, 2021b). In particular, Italy has recorded the second-highest motorization rate (i.e., average number of passenger cars per inhabitant) among the EU countries, preceded only by Luxemburg (Eurostat, 2021b). Environmental psychology research has typically focused on the study of transport behaviors with the main aim to foster behavioral changes in a more sustainable direction, through the identification of key psychosocial and contextual variables (e.g., Steg, 2005; Steg and Vlek, 2009; Donald et al., 2014). As demonstrated by the research literature, while interventions aiming to increase the use of sustainable transport choices *via* economic cues might have a positive effect in the short term, car drivers still prefer to use their car in the long term (Thøgersen and Møller, 2008). Thus, economic incentive alone seems to be insufficient to promote long term sustainable transport choices, which is consistent with the studies focused on other pro-environmental behaviors (e.g., Kaiser et al., 2020). Socio-psychological motives underlying people's transport choices have been widely investigated (e.g., Donald et al., 2014; Manca et al., 2019). However, the knowledge of the narratives supporting such choices in a given place, with its specific and unique features, cannot be obtained through quantitative studies. Furthermore, while the role of the physical environment in influencing people's attitudes, behaviors, and quality of life is well-known (e.g., Gifford, 2014), the effect of public transportation design (e.g., air conditioning, comfort seats, and in-bus features such as Wi-Fi) on travel choices has been substantially overlooked (Lombardi and Ciceri, 2021), even if some studies have highlighted its potential impact by focusing on the attractiveness of the light-rail (Hodgson et al., 2013). Consequently, how do users describe their everyday experience with transport choices? How are these experiences linked to the psychosocial constructs and contextual aspects such as design and aesthetic features of transports?

The present research has been carried out in the specific context of the metropolitan area of Cagliari city (Sardinia, Italy), through a “user-centered” approach (see Gifford, 2002) based on a qualitative-based content analysis strategy. As claimed by different scholars (Schwanen et al., 2011; Thomas et al., 2014), the qualitative methods can provide novel insights to better understand the motivations and views of the people about choices of travel mode, since such methods capture more detailed answers, and, thus, “overcome self-presentation biases and reveal the complexity of motivational structures” (Gardner and Abraham, 2008, p. 188). Hence, this approach provided a deep exploration of the everyday experience of the people with transports in the target place of analysis, i.e., the metropolitan area of the city of Cagliari. In particular, two distinct and complementary studies have been carried out to explore the everyday experience of the people with transport means. The first study looks at the narratives of both car and public transport users, while the second study focuses on the experiences narrated by both the light-rail users and the non-light-rail users.

## Research Rationale

One of the main models used for the prediction of pro-environmental behaviors (Wolske et al., 2017), including transport choices (e.g., Heath and Gifford, 2002; Fu, 2021), is the Theory of Planned Behavior (TPB; Ajzen, 1991), which postulates that the direct antecedent of behavior is behavioral intention, but even perceived behavioral control can trigger such behavior directly (or indirectly through intention). Thus, people's assessment of their ability to perform a given behavior, for instance, in reaching the workplace by bus or by bike, is likely to influence their travel choice.

It has been shown that behaviors may be motivated not only by rational drivers, as postulated by expectancy-value theories such as the Theory of Planned Behavior, but also that affective motives seem to play an important role in influencing travel behavior (Bamberg and Schmidt, 2003; Kals and Müller, 2012) and to implicit attitude toward a sustainable transport (Manca et al., 2019), even though the weight of negative and positive emotions in predicting the intentions to change the way to act has not been clarified so far. The field of emotions is also related to the symbolic factors that may drive people to act in order to widely communicate their own personality, a group membership, or a social status. For instance, the choice to travel with a private and custom car can be due to socio-psychological drivers, such as the desire to highlight personal qualities, group affiliation, or social roles (Gatersleben and Steg, 2013; Moody and Zhao, 2020), and not just for more concrete features of the custom car such as speed and convenience. Among these socio-psychological drivers, social norms have received significant attention as a key dimension in affecting environment-related behaviors and, specifically, travel choices. This kind of social influence includes two types of norms: injunctive norms, which refer to beliefs about others' approval/disapproval of a person's behavior; and descriptive norms, which refer to people's beliefs about others' actual behavior (Cialdini et al., 1991) and represent cues to appropriate behavior for a given situation (Schultz et al., 2008). Both kinds of social norms have proven to have a significant impact on the promotion of sustainable transport choices (e.g., Zhang et al., 2016; Ru et al., 2018). Analytically, injunctive norm was found as a key predictor of public transport use (Thøgersen, 2006), sustainable travel choices (Piras et al., 2021), and car use intention, either in positive (Eriksson and Forward, 2011) or in negative terms (Gardner and Abraham, 2010); whereas descriptive norms emerged as a trigger of the intention to use both public transport and the bicycle (Eriksson and Forward, 2011).

Traveling in a more sustainable way draws attention to the relationship between people and their environment. For instance, sustainable transport choices such as moving on foot or by bicycle could allow a slower and more intense place experience. On a different level, the promotion of public transport use should suggest a specific attention on the appearance of waiting areas, such as bus stops or train/bus stations, whose design features could affect the travel experience of transport users, especially in terms of perceived safety (Loukaitou-Sideris, 1999; Kooi, 2015). In this regard, Abenoza et al. (2018b) have pointed out how particular sites, such as bus shelters and the surrounding environment of bus stops, may influence the perceived safety and crime perceptions of the travelers, which in turn affect the travel mode choice. It was also noted and supposed that the strong relationship between security and overall travel satisfaction (Abenoza et al., 2018a) can influence the intention to use public transport. Therefore, this research has focused on identifying which features of public transport waiting areas trigger people's security perceptions. A literature review by Tucker (2003) reported that lighting, shelters, benches, the surrounding environment and their maintenance, and cleanliness are among the most important features to be considered when designing safe environments related to travel behavior. A recent study pointed out the strong effect of security and economic arguments as significant predictors of positive attitude toward sustainable transport (Manca and Fornara, 2019).

## Objective

The objective of this research is to explore the underlying motives of urban residents' travel choice in a specific target place. Cagliari, located in the south of Sardinia, is the main and most populous city of the island, as well as the port and the main Mediterranean cruise liner hub. Its metropolitan area hosts 17 municipalities with a total of 432.000 inhabitants. Specifically, the aim is to detect directly from the voices of the residents, the barriers and (possible) drivers for more sustainable transport behaviors, in order to better understand how to successfully promote alternative ways of urban mobility rather than using private cars. In fulfillment of this goal, we chose to run qualitative studies based on the focus group technique. In fact, the narrative material from focus group interviews allows to capture those beliefs, attitudes, feelings, and motivations as they naturally emerge from the responses of the speakers. The assumption is that such responses express the (more or less) shared views about the topic which are socially constructed within a given cultural context or social group (Bamberg et al., 2020).

Consistently with our methodological choice, we conceptually relied on a user-centered perspective (Gifford, 2002), which is particularly pertinent to deeply understand the needs of users to identify the main factors that may promote or inhibit sustainable mobility choices.

To date, the metropolitan area of Cagliari provides different choices to travel within it: a cycling network of about 70 km, bike and car sharing services, urban buses that covers the whole city and its suburbs, long-distance buses as links between the city and to other towns or villages of the region, and a recent light-rail system (i.e., a transport system using small trains that operates at a higher capacity and speed, on an exclusive right-of-way) running from the town around a part of its metropolitan area, operating but still with on-going developments (i.e., since April 2015 the light-rail system has been operational on two lines; in 2023 the network expansion will be completed and the inauguration of line 3 is expected).

Specifically, the exploration of views and experiences of the users that are related to different means of urban transport was carried out through two qualitative studies with the same framework but different targets: the first involved general public transport users (Study 1: car users vs. public transport users), the second one had a specific focus on the light rail (Study 2: light-rail users vs. non-light-rail users). Both studies were carried out in the city of Cagliari during the development of the light-rail, connecting various zones of its metropolitan area.

A set of environmental and socio-psychological dimensions were addressed in order to observe their impact in influencing attitudes toward ecological transport. On the basis of the current research literature, we expect an impact of design features (Hodgson et al., 2013), affective motives (Bamberg and Schmidt, 2003; Kals and Müller, 2012), and social norms (Zhang et al., 2016; Ru et al., 2018) on the sustainable travel choices of the users.

## Study 1: Car Users vs. Public Transport Users

### Method

#### Participants

Participants (*N* = 16) were residents in the broad area of the city of Cagliari. To explore the beliefs of the residents who are mainly using different means of transport for their daily journeys, two distinct focus groups were carried out. Specifically, one focus group included public transport users, i.e., those individuals who regularly use public transport (urban and long-distance buses, bike, and light-rail) for most of their daily journeys, while the other focus group included those people who mostly use the private car for their daily moves. The group of public transport users had a total of 6 participants (33.3% men; age range 21–30; M = 25,5), while the group of car users was composed of 10 participants (40% men; age range = 19–33; M = 24,8). Concerning education level, the majority attended senior high school.

#### Procedure and Tool

The focus group technique was used for its suitability in detecting which aspects are the most relevant in influencing the personal travel choice (Clifton and Handy, 2001). Such a technique is based on the discussion of a group of individuals around a set of questions centered on a particular topic or set of topics, and its main objective is to generate conversations that reveal more or less shared opinions concerning a particular issue (Cyr, 2016). The strength point of the focus group material is due to its richness in terms of experiential information, if compared to other data collection methods (Carey and Smith, 1994).

The two focus groups were held by two moderators (i.e., a moderator and an assistant moderator, both co-authors of this paper) in a proper setting (i.e., a room equipped with movable chairs located at the Psychology Building of the University of Cagliari). The duration of each focus group was about 1 h. The moderator welcomed the participants, gave an overview of the topic, and laid out the ground rules. Participants were encouraged to talk spontaneously, and follow-up questions were used to facilitate further discussion of salient issues. The assistant moderator had the role of taking notes and supporting the moderator.

The tool prepared for this study is the focus group interview, which covered an array of 14 questions related to specific aspects—i.e., architectural, functional, and social—related to the transportation experiences. The extent to which each issue was explored was dependent upon its importance on the participants. Questions were based on five macro categories:

1. Advantages and disadvantages of different means of transport [e.g., How would you describe your experience with private (i.e., car and bicycle) and public (e.g., bus) means of transport? What do you think are the main advantages and disadvantages of these different means of transport?];
2. Environmental, personal, and social consequences of different means of transport (e.g., What consequences come to your mind when thinking about the use of different means of transport? For you, personally? For the natural environment? For society as a whole?);
3. Motivations to use their main means of transport (e.g., What are the motivations, for you personally, to use your main means of transport?);
4. Motivations of other people to use different means of transport (e.g., Thinking about other people's choice of transport means, what do you think the main motivation behind their choices could be?);
5. Environmental and architectural elements associated with means of transport (e.g., What comes to your mind when thinking about bus stops/stations/interiors?).

Each focus group was digitally recorded and fully transcribed.

#### Data Coding and Decoding

The content analysis procedure (Krippendorff, 2004) was performed on the two focus group transcripts. Two independent judges coded each focus group discussion following a theory-driven approach, i.e., identifying the relevant sentences and issues related to each topic based on the pre-defined conceptual categories (Hsieh and Shannon, 2005). Such categories concern respectively affect-based and instrumental-based evaluations (Steg, 2005; Manca and Fornara, 2019), perceived behavioral control (Ajzen, 1991), perceived safety (e.g., Ingvardson and Nielsen, 2021; Rahman et al., 2021), social norms (Cialdini et al., 1991; Bamberg et al., 2020), environmental stress (Novaco et al., 1979; Wallenius, 2004), and general environmental and architectural aspects (e.g., aesthetic features). Importantly, people referred to pre-existing categories even if no explicit question about the category was made. For example, the *perceived safety* category has often emerged when asking questions regarding the design features of means of transport.

Based on the aforementioned literature, the judges referred to a concise version of the definition of the construct during the coding process. For example, the affect-based topic was defined as “the extent to which a product is expected to lead to emotional outcomes such as pleasure.” This allowed the judges to focus on the same meaning associated with the constructs of interest.

Each interview transcript was initially split into sentences. An Excel file was preliminarily prepared for this purpose. In this file, each row contained one sentence or unit of meaning. The judges co-constructed this preliminary file, based on the last author's recommendations. In particular, full stops and question marks mainly delimited a sentence; if a participant's opinion was formed by more than one sentence and the sentences together formed one unit of meaning, this case was classified as one sentence only. For example, “*If you have to come to Alghero by car, it's impossible to travel by ARST in Sardinia. For long distances it is impossible*” contains a full stop. However, the sentences are strictly connected to each other in terms of meaning, therefore, it was classified as one sentence. The only exception was when a second participant continued to discuss about the same unit of meaning of a first participant (as it typically occurs in a focus group discussion). In this case, the unit of meaning was separated in two sentences, as different participants contributed to them.

Both judges used the same coding scheme. The scheme was based on the following questions: Does this sentence refer to any of the pre-defined conceptual categories? Which categories? Does the sentence have a positive or negative valence? What is the sentence specifically referring to (i.e., Metro Station, Metro Stops, Metro Tram, Long Distance Buses, Long Distance Buses Station, Railway Station, Urban Bus, Urban Bus Stops, Car, and Setting)? After completing each row, judges answered two more questions: Does the transcript contain any other meaningful category that could not be fully interpreted in light of the pre-existing categories? What does the new category refer to?

Other than categorizing sentences, the data analysis also included the computation of two types of frequencies. The first type only focused on the number of times participants expressed opinions toward socio-psychological factors (i.e., affect evaluations, instrumental evaluations, perceived behavioral control, perceived safety, social norms, and environmental stress) and the environmental, as well as architectural aspects (e.g., design features). For example, the sentence “*The car is like a second home, a second bedroom*” was coded as 1 occurrence of the topic “*Affect-based evaluations*” in Study 1.

The second type of frequency was, instead, computed by counting the number of times that the participants expressed a positive or a negative opinion toward a certain transport mean (e.g., car, bus), or environmental and architectural aspects (e.g., bus station, design features) by type of user. The coding scheme for this type of frequency is presented in Table 1. Taking the same sentence as an example (i.e., *The car is like a second home, a second bedroom*), it was also coded as 1 *positive valence* occurrence for *car*, within the group *car users* of Study 1.

**Table 1:** Example of coding scheme for the second type of frequency computed.

		**Frequency**				
**Valence**	**Object**	**PBU**	**CU**	**LRU**	**NLRU**	**Text**
Positive	Environ. and archit. aspects	–	–	–	–	–
	Bicycle	–	–	–	–	–
	Light-Rail	–	–	–	–	–
	Car	–	–	–	–	–
	Urban bus	–	–	–	–	–
	Long distance buses	–	–	–	–	–
Negative	Environ. and archit. aspects	–	–	–	-	–
	Bicycle	–	–	–	–	–
	Light-Rail	–	–	–	–	–
	Car	–	–	–	–	–
	Urban bus	–	–	–	–	–
	Long distance buses	–	–	–	–	–

**Figure 1:**
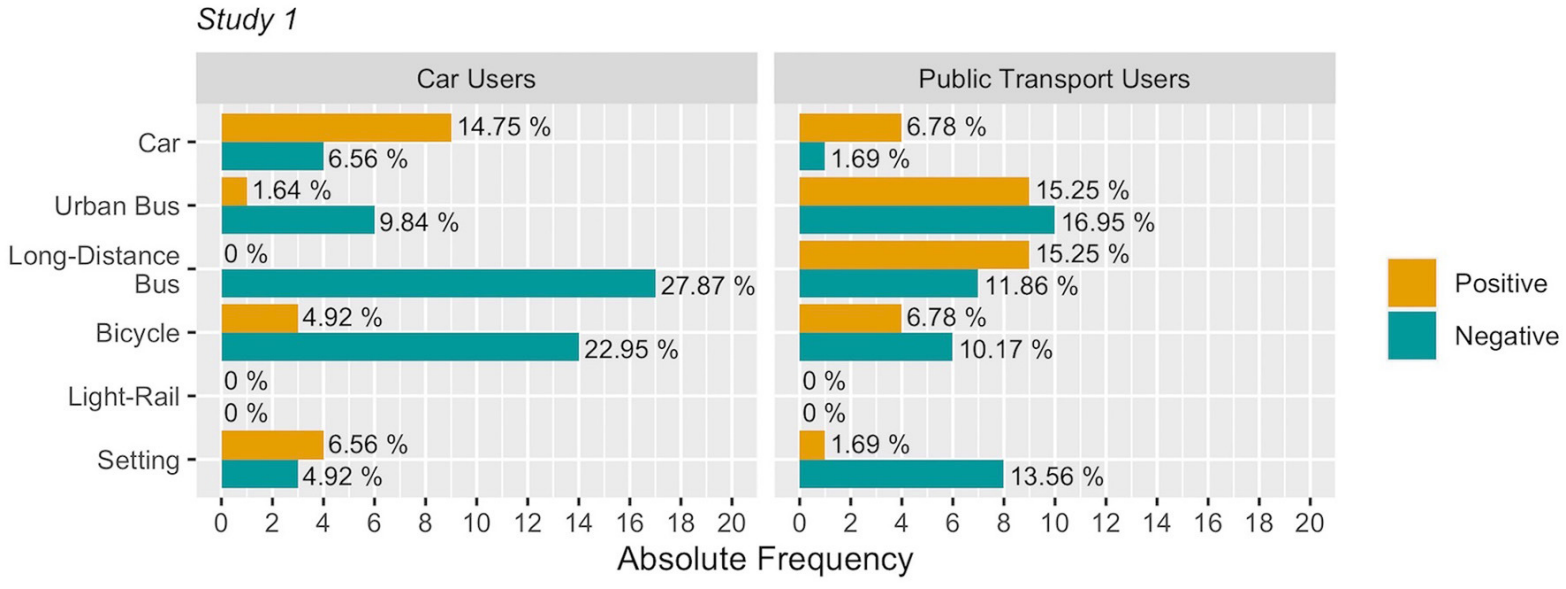
Positive and negative frequencies of sentences associated with different means of transport and with architectural and environmental aspects (i.e., setting). Percentages are computed within each group.

*PBU, public transport users; CU, car users; LRU, light-rail users; NLRU, non-light-rail users; Text, sentence extracted. Figure 1 (Study 1) and Figure 3 (Study 2) present the results based on this scheme*.

The use of the two analysts has allowed to assess the reliability of the coding process (see Golafshani, 2003; Carter et al., 2014). Finally, the two judges compared their content analyses and discussed the inconsistencies until they found an agreement. The degree of consensus/dissensus within each focus group concerning the topics that were dealt with was also analyzed. Participants pointed out an agreement on each addressed issue. Furthermore, as supposed, the choice to set up a group discussion gave the opportunity to each participant to add new details on the topic which were subsequently debated and further shared by the group, given an interesting wealth of content.

## Results

Some trends have emerged, like analyzing the frequency and the valence of the opinions about different means of transport. Specifically, car users have evaluated long-distance buses more negatively than public transport users did (see Figure 1). Furthermore, negative opinions toward the use of the bicycle were expressed over two times more frequently by car users (i.e., 14 occurrences) than those by the public transport users (i.e., 6 occurrences). It is also interesting to note that even though both groups present a similar frequency of negative opinions about urban buses, only public transport users reported a relevant number of positive opinions on urban buses (i.e., 9 occurrences); whereas, car users reported only 1 positive opinion about them. In Figure 1, percentages within groups are also shown. These were computed by initially weighting the raw frequency count by number of participants, then subsequently converting such proportion into percentage. Also note that the interpretation of the results is the same when using either raw frequencies or weighted frequencies.

**Figure 2:**
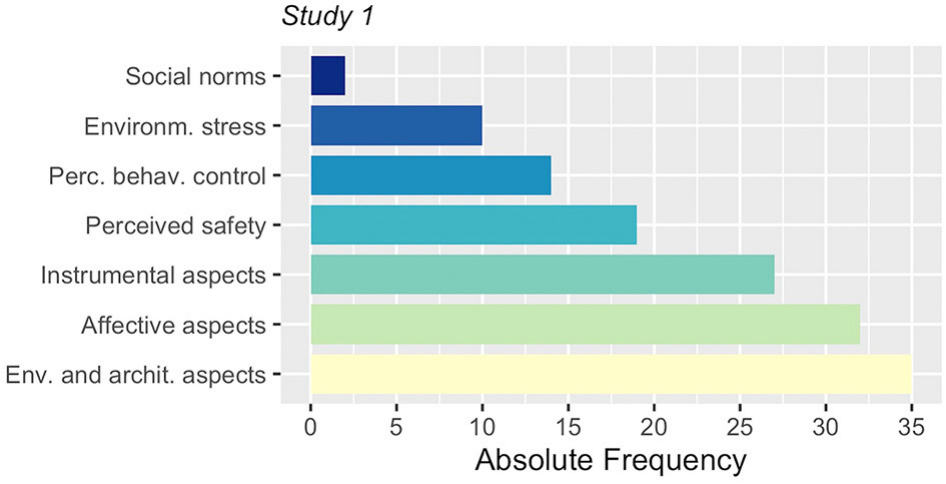
Frequency of socio-psychological, environmental and architectural topics of the group discussions in Study 1.

Narratives on personal travel experience and means of transport choices were mostly focused on affect- or instrumental-based evaluations, respectively, with a total of 32 and 27 occurrences (see Figure 2). Environmental and architectural aspects concerning different means of transport and stations (or stops) consisted of 35 occurrences. The following paragraphs report the summary of the content analysis results for each conceptual category that was considered for this study. In general, discussions were about the selected pre-existing categories and no new category was introduced.

### Affect-Based Evaluations

Participants reported their experiences and opinions by describing how they felt during travel by justifying their travel choices as related to the anticipated feelings. In particular, car users expressed their positive feelings on cars by mainly highlighting the concepts of freedom, independence, and the importance of personal space.

“*(I need) to sit in my own place. I am a little fussy (…), it really annoys me (to be sitting in public transport seats). Furthermore, to be independent. If someone asks me to go out, then I say ok, I just take the car (and drive somewhere).”* Female, aged 25, Car user.

“*(The car) is like a second home, a second bedroom.”* Female, aged 30, Car user.

On the other hand, only the issue of general comfort emerged as a positive feeling associated with car use in public transport users.

Positive emotions about public transport were reported by both groups. The positive emotions concerned the feeling of relaxation derived from the pleasure of being taken to places without driving, the opportunities to look around and to interact with other people.

“*As a positive side there's certainly the social interaction, because it's beautiful: we share those 5 mins the same experience of waiting. So if I find an old woman I help her to take the shopping bag; if you're talking about the university, they see you with the books and ask you what you study. That's already very interesting.”* Female, aged 30, Public transport user.

“*You have the “resignation” that another one will drive. That you don't have to drive.”* Male, aged 25, Car user.

“*I like the idea to look out of the windows rather than drive, instead if you are driving you have to watch the road.”* Female, aged 21, Public transport user.

Similar feelings have been reported about the use of bicycles by both groups. Positive feelings concerned the opportunity to enjoy the surrounding environment. Furthermore, bicycle use has been described as more exciting compared to other means of transport. However, only public transport users reported to also use the bicycle for their daily travels, whereas car users only ride the bicycle for fun during their free time. Feelings of fear related to its use were expressed by car users.

“*We used to ride our bikes and it was a wonderful opportunity to run around Cagliari city.”* Male, aged 30, Car user.

“*I can take a look around if I use the bicycle, instead you have to be more concentrated using the car, so I think that it is nicer.”* Female, aged 21, Public transport user.

Both groups of users defined the bus-station environment as uncomfortable, old, and unsafe.

“*It is uncomfortable. Finding a homeless person with all his stuff on the benches can cause discomfort to a person who is there waiting for his bus/train.”* Female, aged 24, Car user.

“*The bus station is, in general, a bad environment. I have never felt totally safe.”* Female, aged 25, Public transport user.

#### Instrumental-Based Evaluations

Economic factors and travel duration played an important role in the choice of means of transport. Both groups recognized that cars are more expensive means of transport and that public transport requires, usually, a greater travel time. The particular context of Cagliari city, in which the availability of parking places is very limited in the city center, led participants to highlight this problem, thus, motivating Public Transport users toward toward a more sustainable transport.

“*I have to look for a parking spot when I go to San Benedetto Market, it requires too much time by car so taking a bus is faster.”* Female, aged 24, Public transport user.

#### Environmental and Architectural Aspects

Structural environmental aspects of Cagliari city appeared to widely affecting the willingness of people to use the bicycle in the focus group discussions. In particular, the presence of several hills, potholes, and few and inadequate cycle paths have been reported as the main perceived obstacles.

“*The cycle paths are very narrow. There is a narrow cycle path also in Via Paoli that, as you said, people open the car door and kill you.”* Male, aged 21, Car user.

Public transport users expressed a complete dissatisfaction toward structural aspects of urban bus stops. These users defined them as unsuitable to cover people from rain and reported the lack of adequate electronic bus timetables in several bus stops.

“*Yes because in many bus stops there aren't. If it rains, you get wet, you have to wait for the bus and you can't sit down. I think it is a pretty fundamental thing.”* Female, aged 30, Public transport user.

Concerning the aesthetic factors, bus stations for long distance buses were evaluated by both groups as unpleasant and dimly lit. A similar judgment has been reported concerning long distance buses, described as antiquated. Instead, there was no negative judgment about the aesthetic characteristic of urban buses, rather the Public Transport users evaluated them as new and comfortable.

#### Perceived Behavioral Control

Users agree with a sense of disorientation and of waste of time in reference to inadequate structures and services (e.g., lack of electronic timetables) that are supposed to support travel behavior with public transport. The perceived difficulty to use Public Transport has been highlighted by both groups against long distance buses, particularly reporting electronic timetables as too far from bus stops and the impossibility to know in advance the availability of empty seats.

“*There is a board here with tiny timetables that you have to look for. You go there and the first driver you find, you try desperately to ask him which bus you have to take, because you have to go there and you don't understand anything.”* Female, aged 33, Car user.

“*Let's say that what the ARST lacks is perhaps one of those computerized signs with timetables on the inside where the buses stop. To see the timetables, you have to go inside and look at the big board and search for your town. In the meantime, your bus will leave.”* Female, aged 19, Car user.

“*A conception of time gives more security in terms of time and anxiety, because if I know it starts at 1 p.m. for Sassari, I don't if the driver changes his mind, not because he wants it but because there isn't really space, I don't know if I can really take the bus or not. You have different timetables but if you miss one you risk leaving the next day or waiting for the next one, anyway you have to wait four hours. For me personally it makes a big difference.”* Female, aged 24, Public transport user.

Within the Public Transport users, there were mixed opinions concerning long distance buses. In fact, some of them also reported a positive perceived control in talking about the easiness of finding departure time information and reporting it as normally on-time.

“*There aren't bus problems, there are always on time, information is given to you by everybody, both the drivers and the ticket sellers.”* Female, aged 21, Public transport user.

Differently, especially for long distances, cars are a means of transport that are perceived as more reliable and easier to use compared to long distance buses in Cagliari. The perceived ease of bicycle use has been reported as highly compromised by the slopes due to the hilly character of the city.

“*If you have to come to Alghero by car, it's impossible to travel by ARST in Sardinia. For long distances it is impossible.”* Female, aged 24, Public transport user.

#### Perceived Safety

Feelings of security have been reported around all means of transport, except for bicycles by both users.

“*Cycle paths are so narrow, there is also in Via Paoli next to the road, as you said, the drivers open the car's door and they kill you.”* Male, aged 21, Car user.

Bus stations were indicated as unsafe, especially from the evening onwards. Participants reported a lack of adequate lighting and video surveillance in this place. An interesting aspect concerns the relevance of the surrounding environment for the perceived safety. In fact, even though there was a general agreement between users about the perceived safety in travel by buses, participants reported how their feelings of security were affected by the surrounding environment around stations and bus stops.

“*On the bus you are safe, maybe on the street you are not.”* Male, aged 25, Car user.

“*If I see him getting off at the same stop as me, I won't get off.”* Female, aged 25, Car user.

“*(In Piazza Matteotti) the lighting at night would certainly improve a little. If you are around, you can see someone attacking people.”* Male, aged 25, Public transport user.

#### Social Norms

Participants were aware that the willingness to use the bicycle is also affected by perceived social norms that may be different depending on the culture of a country. In particular, car users claimed that in other countries, bicycles are a more common and important means of transport, evaluated as appropriate.

“*We also lack culture because I have been to Germany, Belgium, and England. It's very different there. […] It's completely different here. The bicycle can stay in a very small place.”* Male, aged 30, Car user.

#### Environmental Stress

The main environmental stressor identified by car users was related to the temperature on the public transport. It has been reported that it is usually too high or too low, with the unpleasant consequence of being sweaty or being cold during the journey. Instead, the possibility to regulate the temperature inside the car at will was reported as one of the benefits of using a car from the point of view of the car users. Public transport users focused on the stress caused by traffic that requires high levels of alertness while driving.

“*In fact, I've noticed this: in this period when it isn't that cold, they turn the heaters on at most, so it is a sauna. I swear to you. (…) I was so sweaty. If you want to offer me also a shower service, (…) that is to say, in winter there is a shocking cold instead in summer or in spring they turn the heaters at most.”* Female, aged 33, Car user.

## Study 2: Light-Rail Users vs. Non-Users

### Method

#### Participants

Participants (*N* = 13) were residents in the broad area of the city of Cagliari. Here, the focus was the on-going development of the Cagliari light-rail “MetroCagliari,” hence, light-rail users vs. non-users were distinguished. Following the same methodology of Study 1, two focus groups were held: the group of light-rail users had a total of 7 participants (71,4% men; age range 29–60; M = 41,3), and the group of non-users was composed of 6 participants (50% men; age range 21–49; M = 29). Concerning the education level, the majority attended senior high school.

#### Procedure

The interview was designed following the same structure of Study 1, although different and more specific questions about the light-rail were posed and a new topic was introduced, consistent with the innovation valence as expected for the use of this means of transport. In fact, the new topic concerns the extent to which light-rail users/non-users evaluate transport choices as meaningful for people's status in the society (Steg, 2005; Moody and Zhao, 2020). Data analysis procedure is equal to the one in Study 1.

## Results

**Figure 3:**
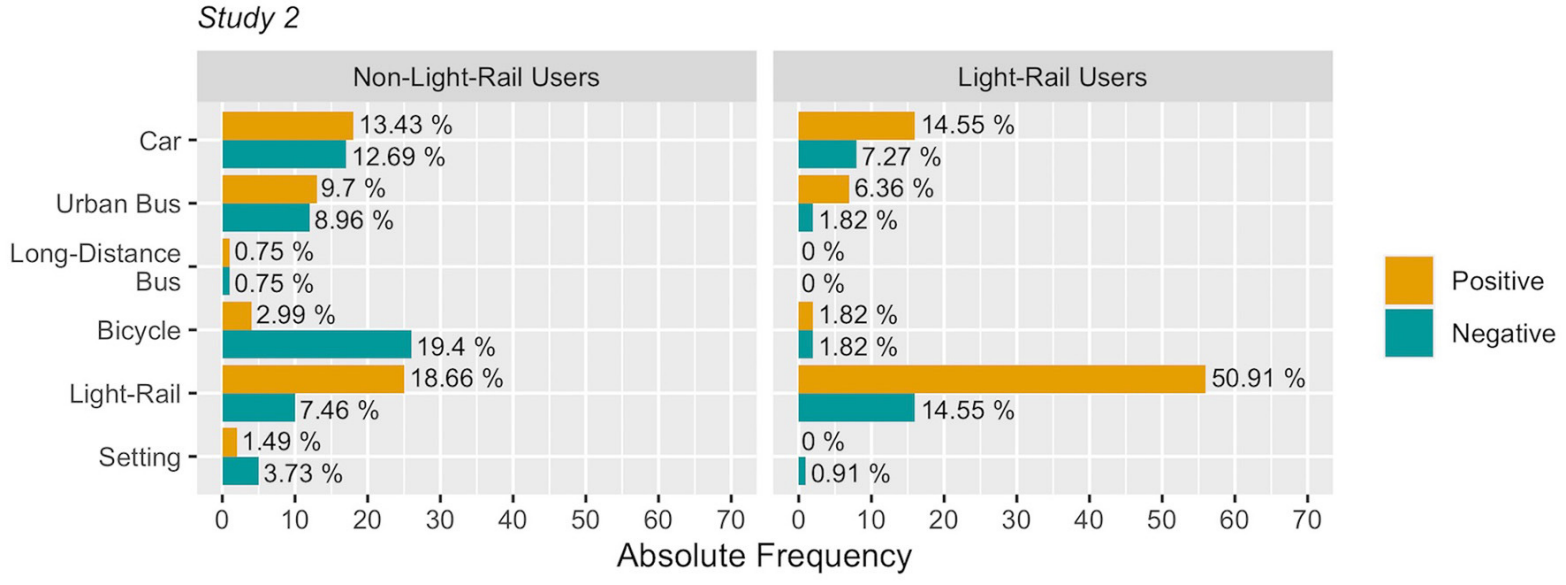
Positive and negative frequencies of sentences associated with different means of transport and with architectural and environmental aspects (i.e., setting). Percentages are computed within each group.

Light-rail users expressed a considerable amount of opinions toward light-rail, with a total of 56 positive sentences and 16 negative sentences (see Figure 3). Opinions toward light-rail by non-light-rail users were, as well, more positive than negative. Instead, opinions toward urban buses by non-light-rail users were well balanced between positive and negative evaluations. The discussion concerning the use of bicycles by non-light-rail users was mostly focused on the negative aspects of it, with a total of 26 negative sentences. The amount of positive and negative opinions toward the car by non-users was similar (Figure 3).

**Figure 4:**
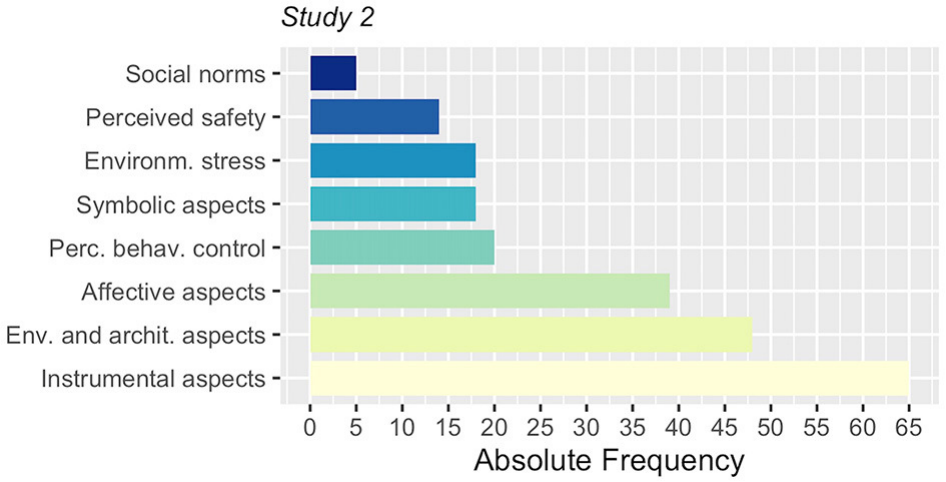
Frequency of socio-psychological, environmental and architectural topics of the group discussions in Study 2.

The motivation underlying the participants' transport choices mainly focused on the instrumental and the affective aspects (see Figure 4). A large part of the discussion has also been reported on the architectural and environmental aspects that are related to transportation.

### Affect-Based Evaluations

The consideration of the car as more than a simple means of transport has been confirmed in the second study by car users. Consistently with Study 1, feelings of freedom and independence related to car use have been repeatedly reported by both users.

“*The car is a very personal environment; a person traveling by car is alone with himself.”* Male, aged 29, Light-rail user.

The valence of the emotions changed when participants focused on the relationship between different drivers. Participants reported nervousness and hatred as negative feelings that arise while driving.

“*In the car, I curse you for not letting me go to the stop.”* Female, aged 23, Non-light-rail user.

“*Another thing can be related to the relationship between people; that is, for example, in the car I always see nervous people as if they were all against each other.”* Female, aged 23, Non light-rail user.

“*The relationship that exists in the car completely changes people. On the contrary, when a cyclist meets another cyclist on the road, they greet each other. In the car people are at their worst.”* Male, aged 43, Light-rail user.

On the contrary, as in Study 1, possible contacts between people during a bus ride seemed to positively contribute to the choice of public transport as means of transport.

“*Taking public transport means to share an environment with other people and this may be appreciated by some.”* Male, aged 29, Light-rail user.

“*I don't say to be also willing to talk, but anyway a smile might be given to a person.”* Female, aged 23, Non-light-rail user.

Non-light-rail users reported a general sense of satisfaction in using bicycles, but, as in Study 1, they also perceived this means of transport as related to free time, especially reporting that the cycle paths in Cagliari were mostly arranged for recreational travels (e.g., beaches, parks).

“*The cycle paths are for pleasure at the moment and not for use: they are placed in places for walking rather than for going to certain places.”* Male, aged 21, Non-light-rail user.

Fatigue and fear were the most mentioned feelings by non-light-rail users during the bicycle discussion.

### Instrumental-Based Evaluations

As in Study 1, economic factors and travel duration motivated the choice between public transport and car use. Cars are perceived as more expensive than public transport, and the latter is perceived as a means of transport that requires greater travel time. The lack of parking areas has also been reported in Study 2 as a motivation to use public transport. Furthermore, participants reported that the use of the light-rail decreases the number of issues related to traffic problems, making the travel time shorter than with other public transport use, thus, increasing the willingness to use the light-rail instead of the car.

“*The light-rail would be much faster than the bus and maybe even the car.”* Female, aged 23, Non-light-rail user.

Light-rail users pointed out a problem related to a lack of integration between urban buses and light-rail services that could potentially limit the use of a more sustainable transport.

“*The lack of integration with the CTM public transport; the fact that there is a public transport's stop at Via Gottardo which it isn't in front of the light-rail and that the “University Express” bus doesn't stop in front of Policlinico: it is ridiculous, despite the fact that there are these two services, there isn't the possibility to arrive quickly coming from Settimo and to continue toward the Brotzu Hospital, because the bus passes under the light-rail without stopping.”* Male, aged 43, Light-rail user.

#### Environmental and Architectural Aspects

The willingness to use bicycles emerged to be compromised, as in Study 1, by inadequate cycle paths and, most of all, by strenuous hills. Non-light-rail users especially stressed these problems, rather than light-rail users who focused more on the inability to get the bicycle inside the light-rail and to use both bicycle and light-rail in the same travel.

“*The light-rail should have more places for bikes because a maximum of two bikes per ride doesn't encourage the use. Only two bikes per ride can be taken.”* Male, aged 30, Light-rail user.

Light-rail stops have been evaluated as inappropriate to cover people from rain and wind and to be isolated from the residential area. This issue has also been stressed in Study 1 concerning urban bus stops. A different opinion has been reported about the internal environment of the light-rail and of its stations. In fact, users reported to be fully satisfied with it, considering the inside of the light-rail to be more comfortable, aesthetically pleasing, and spacious than the urban and long-distance buses, as well as appreciating the light-rail environment of the station.

“*The light-rail is more comfortable because it is larger and more spacious.”* Male, aged 23, Non-light-rail user.

“*I liked the benches, the baskets, the structure.”* Female, aged 49, Non-light-rail user.

#### Perceived Behavioral Control

Users perceived cars as easier to use than general public transport, while the light-rail as easier to be used than urban buses. In fact, light-rails allow users to know exactly when they will arrive at the destination, while the car is described as a means of transport that has the unparalleled feature to be used at any time with large autonomy. Also in this study, the hilly slopes of the city negatively affect the perceived control about bicycle use.

“*(Cagliari) is a city where it is difficult to travel every day by bike.”* Male, aged 23, Non light-rail user.

#### Perceived Safety

As in Study 1, participants declared to feel different levels of safety in the different means of transport, evaluating bicycles as a serious threat for people's life, and describing the surrounding environment of some light-rail stops as dark and consequently less safe. In general, the presence of video surveillance inside a light-rail, its station, and in light-rail stops made these environments safer as perceived by the light-users.

“*(On a bike) you risk your life.”* Female, aged 30, Non-light-rail user.

#### Social Norms

Light-rail users explicitly declared that the choice to use light-rail is affected by perceived social norms. Participants reported that citizens use the light-rail more than the urban buses because it is socially considered as more appropriate. In order to explain that, they justified the choice of using the light-rail as related to a current fashion trend among Cagliari residents. Furthermore, participants reported that seeing people using the light-rail increases chances to use the same public transport by other people.

“*It's a matter of trend, because there is the name in front of the light-rail; because in the end it is a light-rail, it is the same light-rail that was there in 1950s-60s, which is repaired in a modern and comfortable way.”* Male, aged 43, Light-rail user.

“*I think that if more people take the light-rail, more people are interested in taking the light-rail; It is a circular* t*hing.”* Male, aged 30, Light-rail user.

#### Environmental Stress

Only noise pollution, as an environmental stressor, was reported being frequently present in the light-rail. Instead, the environmental stressor identified in the urban bus was related to the feeling of crowding, while stressors for those in cars related to the traffic problem.

“*I've noticed that often on the light-rail there is, this maybe can't be solved, it is too noisy, I mean I continue to hear that sound wuuuufff.”* Male, aged 29, Light-rail user.

#### Perceived Status of Light-Rail Users Stress

Participants focused on the difference between light-rail users and users of other public transport methods such as long-distance buses and urban buses. Light-rail was perceived as a transport used by all walks of life in which even those in high corporate positions use this type of transport. It is important to consider that in Cagliari city, public transport is mainly used by students and less-favored social classes.

“*For example, a friend of mine who brags takes the light-rail; She doesn't take the bus because of the smell, the light-rail is for the elite.”* Female, aged 49, Non-light-rail user.

“*The point of view of some professionals has changed, in fact you sometimes see lawyers, magistrates, and police officers going to Piazza Repubblica by light-rail.”* Male, aged 43, Light-rail user.

“*Those few times I have traveled by bus, I've noticed that there is a slightly different environment, a slightly lower level (than in the light-rail).”* Male, aged 39, Light-rail user.

## Discussion and Conclusion

The present qualitative research sheds light on people's beliefs and attitudes related to the transport choices of residents in a specific target place, i.e., the metropolitan area of the city of Cagliari. Focus group material has allowed the emergence of both cognitive and emotional factors, as barriers or drivers of such choices, that should be considered in order to develop proper strategies for changing a kind of environment-related behavior, such as the use of the private car, that is typically based on habits (Bamberg et al., 2003).

Outcomes of the two studies showed an array of features contributing to the choice of travel behavior and to satisfaction toward the means of transport. (Un)safety, (dis)comfort, and economic aspects emerged as relevant dimensions in influencing the behavior of both usual and potential users of sustainable transport. In this regard, the importance of socio-psychological constructs such as perceived behavioral control and social norms seemed confirmed. Besides, the relevance of design features (such as lighting, space, and architectural elements) in the narratives of the respondents suggests that design issues should be considered in promoting public transport, which represents a more sustainable travel mode than the private car. Improving the design features could also exert a positive influence on symbolic and affective values related to public transport choices. To date, no have investigated if and how these different pathways may interact in the field of public transport choices. From the present study, pleasant aesthetic features, along with modern and cutting-edge means of transport, were associated with people holding higher status positions in society, compared with those from unpleasant aesthetic features. Future studies should investigate this possible interaction since symbolic outcomes associated with car use have been shown to be extremely important (e.g., Lois and López-Sáez, 2009; Moody and Zhao, 2020). We propose that promoting symbolic outcomes could (i) mitigate the effect of those symbolic outcomes associated with car use, and (ii) attract more car users to explore the use of public transport. The importance related to the general symbolic factors on travel mode has also been pointed out by Murtagh et al. (2012a) from an identity-based perspective. The authors showed that the degree to which people identified themselves with a certain social category (e.g., parents, workers, motorists) has influenced their willingness to use different means of transport (i.e., car and public transports). In this sense, the authors concluded that multiple and competing identities can influence the transport choice decision, and that the centrality and the salience of a certain identity can push the preference of an individual of his/her travel means. In another study, Murtagh et al. (2012b) have also shown that changes in travel mode could be perceived as a threat toward identity motives (i.e., self-esteem, generalized self-efficacy, continuity, and distinctiveness). In interpreting the current research results in light of Murtagh's work, public transport design features and efficiency improvements may support people's identity motives such as self-esteem and self-efficacy, thus, resulting in an easier transition toward more sustainable transport means.

Likewise, the development of modern and cutting-edge means of transport such as light rails has been shown to contribute to a positive “place of image” of the city (Ferbrache and Knowles, 2017). Therefore, the implementation of new means of public transport needs to consider the consequences associated with cultural and identity aspects of the citizens and of the city as a whole. While opinions toward light-rails were mostly positive in the present research context, the temporary disruption in the streets during the installation phase of light rails, along with changes of the general image of the urban environment, could potentially and negatively interfere with people's place attachment (e.g., Anton and Lawrence, 2016; Von Wirth et al., 2016; Clarke et al., 2018; Reese et al., 2019).

Consistently with other research findings (see Ellaway et al., 2003), a higher perceived security is associated with the use of a private car, whilst, on the other hand, public transport is viewed as unsafe. The necessity of individuals to protect their own personal space (see Hall, 1966) appears to be particularly salient in transport choices, as already noted in previous literature (Mann and Abraham, 2006). All respondents reported that both the presence of crowds and the narrowness of the settings decrease feelings of protection, and, consequently, the use of public transport.

With concern to the role of architectural elements in enhancing the perception of security, this research confirms the evidence which emerged in other settings (e.g., the stadiums: Manca and Fornara, 2015). More specifically, spatial and physical features were indicated as crucial in providing a positive travel experience. The proper lighting of stations, waiting areas, bus stops, and vehicles emerged as strongly related to higher security and satisfaction levels in the narrative of the respondents. In particular, most women showed feelings of fear and anxiety related to journeys in places that are poorly lighted, thus, preferring the use of a private car in reducing the potential risks of being assaulted. Enhancing the overall perceived behavioral control of the users seems to promote a better opinion on sustainable transport, thus, confirming the salience of this pattern for the promotion of pro-environmental responses (e.g., see Wolske et al., 2017; Fornara et al., 2020). The access to an interactive service information (e.g., electronic signs, timetables, routes) was mentioned as a key element for reducing the uncertainty related to the waiting time and the real transit of the bus. Therefore, architectural elements constitute a crucial aspect in influencing attitudes toward sustainable transport and travel choices. These outcomes suggest that design features should be taken more into account in the planning of both external environments (i.e., stations and bus stops) and of the interior of the public vehicle to increase the safety of the passengers, and, consequently, to promote the use of a travel mode that is an alternative to the private car.

The importance of economic arguments in orienting transport choices is another point that emerged from focus group narratives, thus, confirming what was found in other studies (Wardman, 2001; Jakobsson et al., 2002; Manca and Fornara, 2019). More specifically, people show a biased perception of their travel cost, since public transport tickets were valued as too expensive when compared with the fuel cost of the private car. In this regard, a positive influence on attitudes toward public transport has been pointed out in studies that tested the potential role of free tickets (Fujii and Kitamura, 2003; Ayako and Satoshi, 2007), underlining the persistent use of buses after the free period. Thus, economic incentives should be implemented together with the provision of a comfortable and safe transport service.

The pattern of frequency related to psychosocial and contextual variables associated with transport means was consistent among the two studies. In particular, instrumental, affective, environmental, and architectural aspects were the most frequent topics discussed, while social norms were the less frequent topic. The latter corroborates with studies addressing the role of people's awareness of social influence on their everyday choices. In fact, people tend to underestimate the effect of social norms on their individual behavior, despite norms being one of the strongest behavioral predictors (Nolan et al., 2008).

This study presents several limitations. First, participants consisted of adults and young adults, future studies should also consider the elderly who might present different needs and experiences. Second, the educational level of our samples was similar. All participants had at least a high-school diploma. Therefore, our findings may be affected by socio-demographics. Another limitation concerns the transferability of the findings. We suggest these findings to be relevant for similar social contexts where social norms strongly sustain car use. For example, narratives among car users and public transport users may include different contents when considering citizens living in Amsterdam (i.e., where bicycle use is very common). Moreover, large municipalities offer multiple and various transport options such as feasible mobility services (e.g., Uber, subway), which were not available in the context of the present study. A further limitation concerns the novelty associated with the implementation of the light-rail in the present context of study. In fact, future studies should verify if and how people's opinion toward new public transport developments may differ according to the installation phase of the project. In fact, Ferbrache and Knowles (2017) highlighted the importance of carrying out longitudinal research to capture the changes in people's narrative in the long-term in similar research contexts.

In light of designing new spaces such as waiting areas, bus stops, and means of transport, these findings highlight a set of users' needs that could increase the use of sustainable travel choices such as public transport. Opting for strategies that improve the user experience in public transports might be particularly useful for those lacking in environmental motivations to use public transport (e.g., De Groot and Steg, 2009). In conclusion, by employing a user-centered approach (Gifford, 2002) and evidence-based guidelines (Hamilton, 2003), the present study sheds light on actual and potential experience, preferences, and needs of the users that are related to sustainable travel choices.

## Data Availability Statement

The data analyzed in this study is subject to the following licenses/restrictions: Qualitative study with focus groups recording in Italian language. Requests to access these datasets should be directed to Sara Manca, saramanca@email.it.

## Author Contributions

SM: contributed to conceptualization, methodology, investigation, data analysis, and first-draft writing. FT: conceptualization, methodology, investigation, data analysis, and writing (reviewing). NM: methodology, investigation, and data analysis. FF: conceptualization, methodology, and writing (reviewing). All authors contributed to the article and approved the submitted version.

## Funding

This research was funded by the Autonomous Region of Sardinia, Regional Law n.7/2007, FSC 2017, Grant Number RASSR78308.

## Conflict of Interest

The authors declare that the research was conducted in the absence of any commercial or financial relationships that could be construed as a potential conflict of interest.

## Publisher's Note

All claims expressed in this article are solely those of the authors and do not necessarily represent those of their affiliated organizations, or those of the publisher, the editors and the reviewers. Any product that may be evaluated in this article, or claim that may be made by its manufacturer, is not guaranteed or endorsed by the publisher.
